# *B.infantis* enhances immunotherapy for Guillain-Barre syndrome through regulating PD-1

**DOI:** 10.1186/s12883-022-03046-w

**Published:** 2023-01-28

**Authors:** Peng Shi, Di Nian, Hongdang Qu, Ming Ye, Chun Wang, Li Li, Qian Li

**Affiliations:** 1 Department of Neurology, first Affiliated Hospital of Bengbu Medical College, Bengbu, 233004 Anhui China; 2 Department of Medical Examination, Bengbu Medical College, Bengbu, 233030 Anhui China

**Keywords:** Guillain-Barré syndrome, *B. infantis*,*e*xperimental autoimmune neuritis animal model, T helper cells, PD-1

## Abstract

**Background:**

Guillain-Barré syndrome (GBS) is a rare, autoimmune disease. *B.infantis* is reported to be effective in alleviating GBS by regulating abnormal function of T helper (Th) cells.

**Objectives:**

In this study, T cells were isolated from healthy and GBS patients. The therapeutic effect of *Bifidobacterium infantis* (*B.infantis)* and whether it is achieved by PD-1 was examined at cellular and animal models.

**Methods:**

We used CCK-8, flow cytometry and real-time PCR to determine the differentiation of T cell subsets at cellular level. Then, an experimental autoimmune neuritis (EAN) animal model using six-week SD rats (*n* = 30, male) weighing 180–200 g was established to support the role of *B. infantis* in GBS through PD-1.

**Results:**

*B. infantis* inhibited the proliferation and promoted apoptosis of T cells from GBS. At the same time, the expression levels of PD-1 increased, which was correlated with decreased T-bet (Th1) and ROR-γt (Th17) and increased Foxp3 (Treg) expression. Moreover, *B. infantis* alleviated the symptoms of GBS. Th1 and Th17 cells decreased while Treg cells increased after *B. infantis* treatment, which could be partly abrogated by PD-1 inhibitor.

**Conclusions:**

We concluded from this study that *B.infantis* alleviated GBS partly through PD-1.

**Supplementary Information:**

The online version contains supplementary material available at 10.1186/s12883-022-03046-w.

## Introduction

Guillain-Barre syndrome (GBS) is an autoimmune disease involving the peripheral nervous system with high disability and mortality. GBS is the commonest post-infectious inflammatory peripheral neuropathy with undiscerned aetiology. The commonly reported antecedent infections implicated include *Campylobacter jejuni*, chikungunya, dengue, and *Japanese encephalitis* [1]. The commonly used treatments included plasma exchange and immunosuppressive therapy. However, no treatment can block the progress of the disease. Therefore, the pathogenesis, early diagnosis and effective therapy have become the hot field. At the same time, the experimental autoimmune neuritis (EAN) rat model, as the classic animal model of GBS, has been widely recognized due to similar main pathogenesis, pathophysiology and histological changes to acute inflammatory demyelinating neuropathy (AIDP) and acute motor axonal neuropathy (AMAN) of GBS.

Recently, with the development of molecular biology and immunology, it has been found that there are a large number of microorganisms in the human intestinal tract,which closely related to substance metabolism and immunity [2, 3]. Under normal circumstances, the intestinal flora and the host maintain a dynamic balance, and play an important role in the regulation of innate and adaptive immune responses and intestinal barrier homeostasis [4–6]. Once the flora is out of tune and the balance is destroyed, the pathophysiological state of the host may be affected through immune regulation, metabolism and other pathways [7, 8]. Studies have shown that intestinal flora imbalance and structural changes is not only related to intestinal inflammatory diseases, but closely related to chronic diseases such as autoimmune diseases, diabetes, senile dementia and obesity [9–12]. Moreover, more and more evidences show that probiotics can regulate the host immune system and inhibit abnormal autoimmune responses. An important study found that in vivo and in vitro intestinal *Bifidobacterium infantis* (*B. infantis)* subgroup has a great influence on the differentiation of CD4^+^T cell and as the most important and the most common intestinal probiotics group, has played an important role in the treatment of autoimmune inflammatory intestinal Crohn’s disease. Its action mechanism included enhancing the intestinal mucosal barrier; regulating the balance of intestinal flora against pathogenic bacteria; inducing and activating non-specific and specific immune responses [13, 14]. Increasing the level of intestinal *Bifidobacterium* in infected mice can reduce intestinal autoimmune inflammation mediated by intestinal Th17, such as intestinal mucosal damage, diarrhea and weight loss [15]. *Bifidobacterium* also altered the composition of the gut microbiota systematically in a regulatory T cell (Treg)-dependent manner. Moreover, this altered commensal community enhanced both the mitochondrial fitness and the IL-10-mediated suppressive functions of intestinal Tregs [16]. Programmed death 1 (PD-1) is an inhibitory receptor on T cells and its ligand is programmed death ligand 1 (PD-L1). Ding et al. showed that PD-L1 treatment inhibited lymphocyte proliferation and altered T cell differentiation by inducing decreases in IFN-γ^+^CD4^+^ Th1 cells and IL-17^+^CD4^+^ Th17 cells and increases in IL-4^+^CD4^+^ Th2 cells and Foxp3^+^CD4^+^ regulatory T cells [17]. In addition, immune checkpoint inhibitors such as anti-PD-1 and anti-PD-L1 are associated with a higher risk of neurological complications such as GBS [18].

In summary, *B. infantis* can interfere with the occurrence, development and outcome of some autoimmune diseases through affecting the function of T cells, but how it affects T cell function is still unclear. Our previous studies have found that [19, 20], gastric administration of *B. infantis* can improve intestinal microecological imbalance and the autoimmune inflammatory reaction through regulating the level of Th17/Treg cells in EAN animal model. In this study, we focused on the relationship between *B. infantis* and PD-1 signaling.

## Materials & methods

### Participants

Fifteen patients with GBS were recruited sequentially at the Neurology Department, and 15-matched healthy controls were recruited at the Physical Examination Center of First Affiliated Hospital of Bengbu Medical College from Jan 1st 2020 to Jan 1st 2021. The GBS was diagnosed, according to the international diagnostic criteria [21] including an acute progressive symmetrical weakness of the extremities with areflexia or hyporeflexia, albuminocytological dissociation in the CSF, and demyelinating/axonal neuropathy by electrophysiology. Albuminocytological dissociation was defined as abnormal levels of proteins with a total cell count of ≤10/mm^3^ in CSF [21]. Individual patients were excluded if she/he had a history of autoimmune diseases. Written informed consent was obtained from individual participants, and the experimental protocol was approved by the Ethical Committee of First Affiliated Hospital of Bengbu Medical College.

### Lymphocytes cells isolation

Peripheral blood mononuclear cells (PBMCs) (10 ml) were isolated using Ficoll-Hypaque solution (Haoyang biotech, Tianjing, China), and cultured in RPMI-1640 at 37 °C in 5% CO_2_ for 2 h until the monocytes adhered to the bottom of culture flasks. Then the lymphocytes suspended in middle were isolated. The purity was more than 90% through flow cytometry assay and the viability was more than 90% through Trypan blue staining. Cells were cultured at 37 °C in RPMI 1640 medium, supplemented with penicillin-streptomycin prior to the analysis.

### CCK-8 assays of cell viability

The effects of *B. infantis* on proliferation of T cells were examined using Cell Counting Kit-8 assays (CCK-8, Dingguo, Beijing, China). T cells (1000 cells/ well) from GBS (*n* = 15) were divided into five groups including control, GBS, GBS + *B. infantis* (10^7^ cfu /ml), GBS + PD-1 inhibitor G4C2 (10 μg/ml) and GBS + *B. infantis* (10^7^ cfu /ml) + G4C2 (10 μg/ml), then maintained at 37 °C for 24, 48, 72 and 96 hours [22]. G4C2 was a rabbit monoclonal antibody against PD-1.

### Flow cytometry

The effects of *B. infantis* on apoptosis of T cells were examined using Flow cytometry. The T cells were adjusted to 5 × 10^6^/ml. After fixation of the cells with 4% paraformaldehyde and permeation with Triton X-100, both for 5 min, PI was added and incubated at 37 °C for 30 min. The supernatant was removed by centrifugation, and the cells were washed 3 times with PBS. The ratio of the two parameters was calculated and analyzed using a FACSCalibur flow cytometer (Becton Dickinson, NJ, USA).

### Western blot

Total protein extraction was obtained using high-strength RIPA buffer (100 μl). The proteins were separated by 12% SDS-PAGE and then transferred to polyvinylidene difluoride membranes (PVDF). After blocking the membrane with 5% nonfat milk for 1 h at room temperature, the membrane was incubated with rabbit anti-PD-1 (Abcam, Shanghai,China, 1:1000) and rabbit anti-GAPDH (Abcam, Shanghai, China, 1: 1000) overnight at 4 °C. After 6 washes with TBST (containing 1% Tween 20), 5 min each time, the membranes were incubated with an anti-rabbit secondary antibody conjugated to horseradish peroxidase (Abcam, Shanghai, China, 1: 500) for 1 h at room temperature. The membranes were washed with TBST and developed using a Tanon 5200 chemiluminescence image analysis system (Tanon, China) [23].

### Real-time polymerase chain reaction (PCR)

CD4^+^T cells (10^7^ cells) from healthy control (*n* = 15) and GBS (n = 15, were treated with or without 10^7^ cfu /mL *B. infantis* and total RNA from CD4^+^T cells of different groups were extracted using Trizol (Invitrogen, CA, USA) and a reverse transcription was carried out with a Reverse Transcriptase kit (Takara, Dalian, China). Real-time PCR was performed with the following primers: T-bet (sense 5′--GGACCCAACTGTCAACTGC-3′, anti-sense 5′-TGTCGCCACTGGAAGGA-3′); GATA-3 (sense 5′-GCCATTCGTACATGGAAGC-3′anti-sense 5′-CGGAGGGTAAACGGACAG AG-3′); RORC-γτ (sense 5′-GCAGCAACAGGAACAAGTGG-3′, anti-sense 5′-GCTTTGCCTCGTTCTGGACT-3′) and Foxp3 (sense 5′-GCAGCAACAGGAACAAGTGG-3′, anti-sense 5′-GCTTTGCCTCGTTCTGGACT-3′); β-actin (sense 5′-GTGGACATCCGCAAAGAC-3′, anti-sense 5′-AAAGGGTGTAACGCAACTAA-3′). PCR was carried out in a 7300 real-time PCR System (Applied Biosystems, CA, USA) using general SYBR green fluorescence detection for 10 min at 94 °C followed by 45 cycles of 15 s at 95 °C, 30 s at 60 °C and 30 s at 72 °C. Relative quantitative expression was calculated as 2^-ΔΔCT^ methods.

### EAN animal model

Six-week SD rats (*n* = 30, male) weighing 180–200 g were purchased from experimental animal center of Bengbu Medical College. The mice were housed in a specific pathogen-free (SPF) environment with a light:dark cycle of 12:12 h at 25 °C with a humidity of 50–60%, and they were free to drink and eat daily. All procedures of animal handling were carried out in accordance with the protocols of the animal care guidelines of the Institutional Animal Care and Use Committee of Bengbu Medical College. The animal study protocol number was [2020]242. Establishment of the EAN rat model has been described previously [19]. The animals were randomly divided into 5 groups (*n* = 6 each) including the Control group, Model group, Model+ *B. infantis,* Model*+*PD-1 inhibitor G4C2 and GBS+ *B. infantis +* G4C2*.* The animals were kept in separate cages with clear labels to avoid confounder. All the animals included in the analysis.

In brief, EAN rats were induced by subcutaneous injection at hind feet with 100 μL of an emulsion. The emulsion containing 200 μg P0_180–199_ peptide (KE Biochem Shanghai, China), 1 mg Bacillus Calmette–Guérin vaccine (Wanma pharmaceutical co., LTD, Zhejiang, China), and 1 mg *M.tuberculosis* (Huayun, Guangzhou, China) in 100 μL. Rats were scored every 5 days after immunization for development of EAN as follows: 0 = normal, 1 = limp tail, 2 = mild paresis of the hind limbs, 3 = severe paraparesis or paraplegia of the hind limbs, and 4 = tetraparesis. Animals were randomly divided into five groups, including the control group, the model group, the *B. infantis* (10^9^ CFU/ml) and the *B. infantis* (10^9^ CFU/ml) + PD-1 inhibitor G4C2 (3 mg/kg, Junshi Biological Medicine Co., LTD, Shanghai, China). Each group contained six rats. *B. infantis* prescription started at week 2 after immunization. *B. infantis* was given intragastric administration and G4C2 was given intraperitoneal administration. Mice were fed with 10^9^ CFU/ml of *B. infantis* every day and lasts until day 25 after immunization. Blood samples were collected for T cell differentiation assay at day 25 after immunization. At the end of the study, the animals were injected isoflurane overdose and given cervical dislocation to sacrifice the animals.

### Electrophysiological examination of sciatic nerve

The rats were anesthetized by administering intraperitoneal injection of pentobarbital sodium (30 g/L) as 45 mg/kg. After complete anesthesia, the skin was cut between right biceps muscle of thigh and semimembranosus muscle with blunt separation to expose the sciatic nerve. One cm long sciatic nerve was taken and fixed in 2.5% glutaraldehyde. After being washed with PBS, it was dehydrated in series, and treated with absolute ethanol for 15 min, and 95% acetone for 15 min. Then it was treated with anhydrous acetone for 10 min, and the solution was changed once every 5 min. The tissue was placed in the embedding agent propylene oxide solution (1: 1) for 1 hour, and in the pure embedding agent for 3 hours. Its ultra-thin sections were double-stained with uranyl acetate and lead citrate. They were observed and photographed with a transmission electron microscope (TEM) [19].

### Statistical analysis

Each experiment was repeated more than 3 times, and SPSS18.0 statistical software was used for statistical analysis. All test results were expressed as Means ± SE, and *t* test or ANOVA was used for the data comparison, with statistical significance when *p* < 0.05. Spearman rank correlation test was used to evaluate the relationship between variables. *p* value < 0.05 was considered statistically significant.

## Results

### *B. infantis* promotes the protection through PD-1

**Fig. 1:**
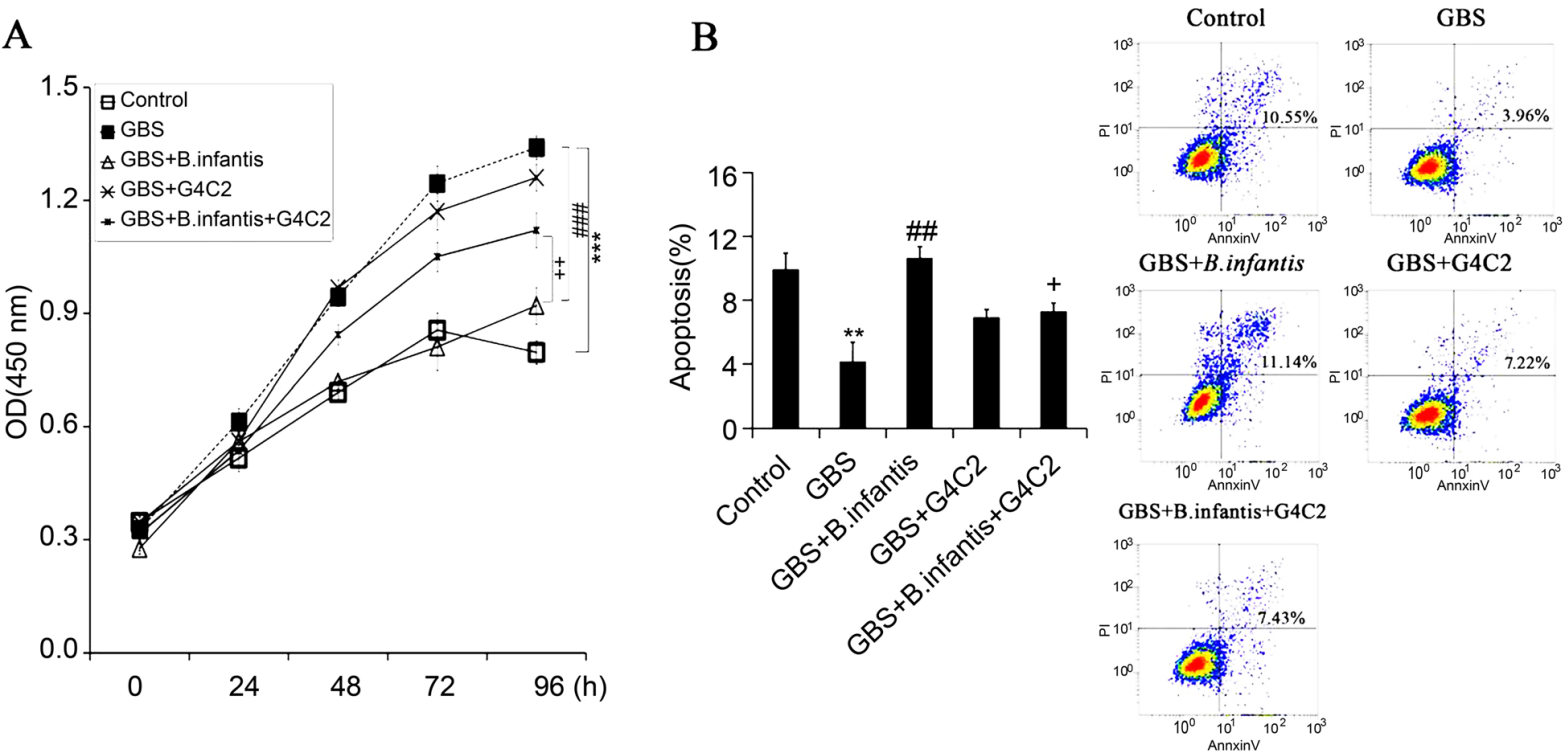
*B. infantis* inhibited the proliferation and promoted apoptosis of T cells from GBS patients through PD-1 (*n* = 5). **A**, cell proliferation was assayed using CCK-8 assay; **B**,cell apoptosis was assayed using flow cytometry. The experiments were repeated 5 times. ** *p* < 001 and *** *p* < 0.001 vs. Control; ^##^ p < 001 and ^###^ *p* < 0.001 vs. GBS;^+^ *p* < 005 and ^++^ *p* < 0.01 vs. GBS+ *B. infantis*

We isolated T cells from healthy and GBS patients to examine the protection of *B. infantis* on proliferation. The demographic and clinical characteristics of participants were listed in Table 1.The results showed that T cells from GBS patients showed exaggerated proliferation. The proliferation rate of T cells was over 90% compared with the control group. *B. infantis* inhibited the proliferation and brought proliferation close to normal levels. PD-1 inhibitor G4C2 could partially reverse *B. infantis*-induced inhibition of proliferation (Fig. 1 A). On the other hand, T cells from GBS patients showed inhibiting apoptosis. Compared with the normal group, the inhibition rate was 60%. *B. infantis* promotes the apoptosis of T cells and made apoptosis close to normal levels. The pro-apoptotic effect of *B. infantis* was partly inhibited by G4C2 (Fig. 1 B). These results suggest that *B. infantis* can influence T cell proliferation and apoptosis through PD − 1 signal.

**Table 1:** the demographic and clinical characteristics of participants

	Healthy controls	GBS patients
Number	15	15
Age	37.8 ± 6.9	35.4 ± 9.6
Gender (Male)	14	15
WBC in CSF (10^6^/L)	0.85 ± 0.12	1.16 ± 0.31
WBC in plasma (10^9^/L)	5.37 ± 3.16	6.88 ± 2.55
Albumin in CSF(g/L)	0.29 ± 0.08	0.73 ± 0.31**
Albumin in plasma (g/L)	48.63 ± 5.12	38.65 ± 2.53

**p < 0.01 vs. HC

**Fig. 2:**
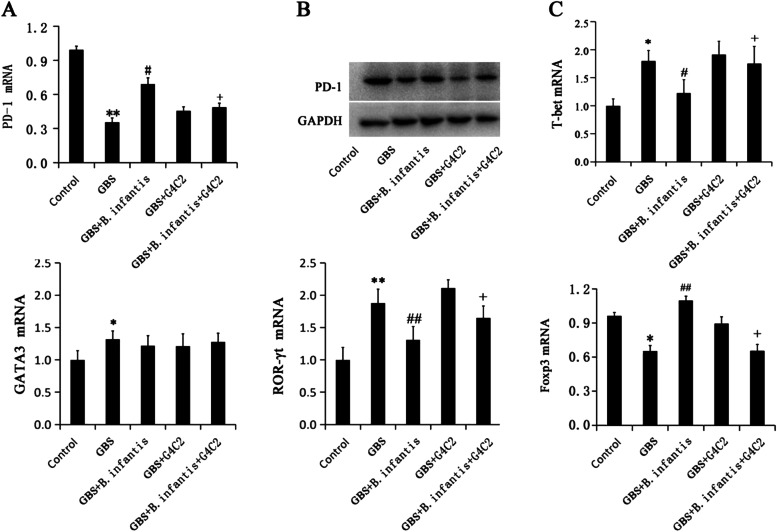
*B. infantis* inhibited the abnormal differentiation of T cells from GBS patients through PD-1 (n = 5). **A**, the expression levels of PD-1 was assayed using RT-PCR; **B**, the protein expression levels of PD-1 on T cells were assayed using Western blot; **C**, the expression levels of T-bet, GATA3, RORγt and Foxp3 on the T cells were assayed using real-time PCR. The experiments were repeated 5 times. * *p* < 005 and ** *p* < 0.01 vs. Control;^#^ *p* < 005 and ^##^ *p* < 0.01 vs. GBS;^+^ *p* < 005 vs. GBS + *B. infantis*

At the same time, the mRNA and protein expression of PD-1was significantly decreased in Model group. *B. infantis* could induce the increases of the mRNA and protein expression of PD-1, which could be abrogated by PD-1 inhibitor G4C2(Fig. 2 A,B) . So far, it proved that there are four different subsets of CD4^+^T cells-Th1, Th2, TH17 and regulatory T cells (Treg). T-bet is the transcription factor specific for inducing Th1 cell differentiation. GATA3 induces Th2 cell differentiation. ROR-γt induces the differentiation of Th17 and Foxp3 induces the differentiation of Treg [24]. Using real-time qPCR, we found that the T cells from model group animal showed enlarged expression of T-bet, GATA3 and ROR-γt. The expression of Foxp3 was significantly decreased. After *B. infantis* treatment, the T cells expressed less T-bet and ROR-γt and more Foxp3 and PD-1 on T cells,which could be abrogated by PD-1 inhibitor G4C2 (Fig. 2 C)

### *Infantis* inhibits GBS via. PD-1

**Fig. 3:**
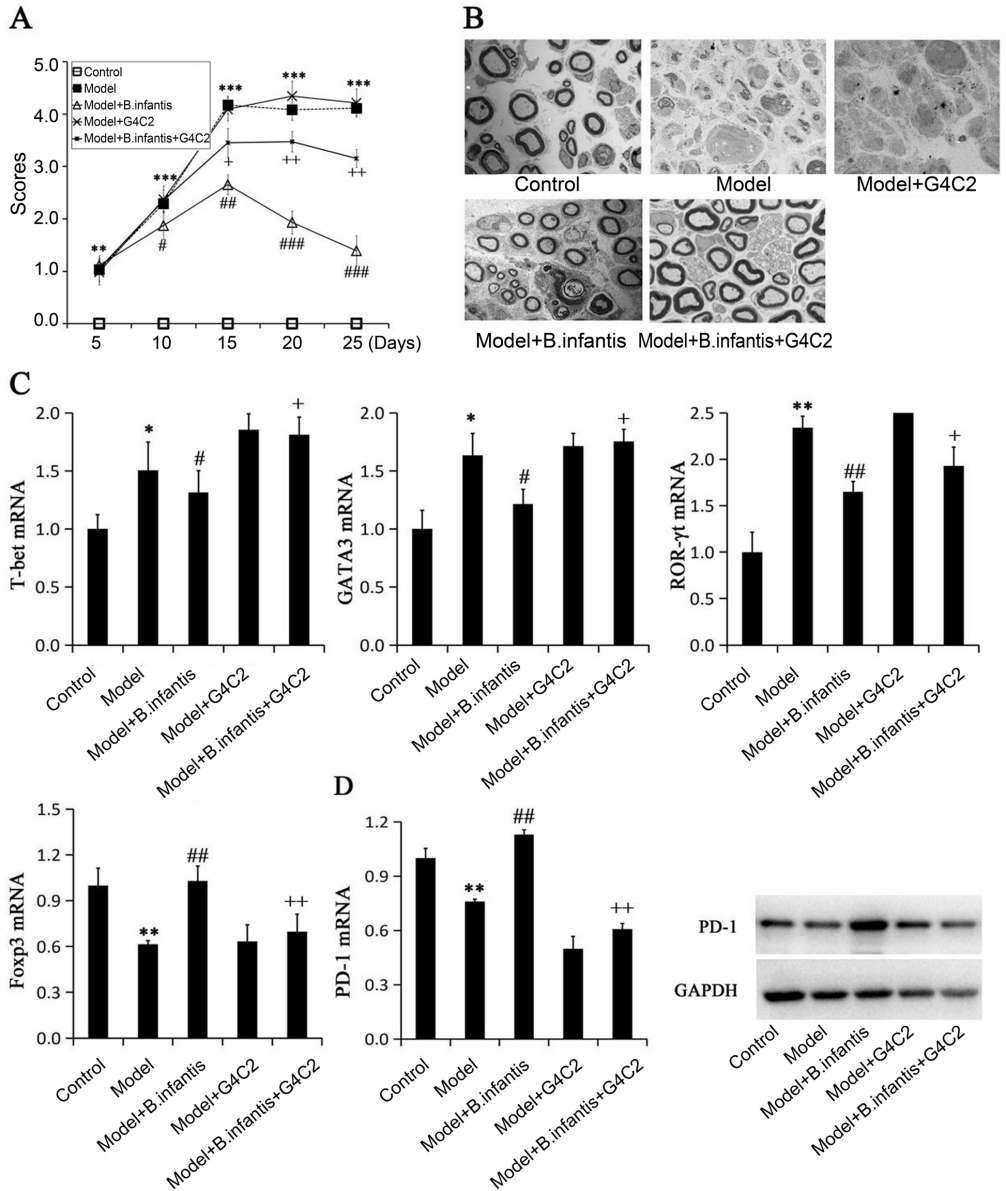
*B.infantis* inhibits GBS through upregulating PD-1*in vivo* (*n* = 6). **A**, the clinical scores of animal models; **B**, the demyelination of the sciatic nerve was assayed by TEM. **C**, the expression levels of T-bet, GATA3, RORγt and Foxp3 on the T cells were assayed using real-time PCR; **D**, the mRNA and protein expression levels of PD-1 on T cells were assayed using real-time PCR and Western blot. Six animals were divided into each group. * *p* < 005, ** *p* < 0.01 and *** *p* < 0.001 vs. Control; ^#^ *p* < 005, ^##^ *p* < 0.01 and ^*###*^ *p* < 0.001 vs. Model;^+^ *p* < 005 and ^++^ *p* < 0.01 vs. GBS+ *B. infantis*

No animals died in all groups. The rats in the EAN group began to exhibit clinical symptoms from the first day after immunization and exhibited tail mopping behavior on the 5th day. The clinical scores were 4.168 ± 0.38 on Day 15 and it stayed above 4, indicating that the modeling is successful. The clinical scores were significantly improved after administration of *B. infantis* from the 10th day. The clinical scores were 2.413 ± 0.44 on the 10th day and then decreased to 1.15 ± 0.32 on the 25th day. The protective effects of *B. infantis were* partly reversed by PD-1 inhibitor G4C2 (Fig. 3 A). In addition, compared with the control group, obvious demyelination was observed in the EAN group, which was alleviated after administration of *B. infantis*. The process was partly reversed by PD-1 inhibitor G4C2 (Fig.3B).

After *B. infantis* treatment, we found that T-bet and RORγt cells were significantly lower than those in the model group, while Foxp3 increased, which was abrogated by PD-1 inhibitor G4C2 (Fig. 3 C). Moreover, the protein and mRNA expression of PD-1 in the model group was significantly decreased, which could be enhanced by *B. infantis* treatment. PD-1 inhibitor G4C2 could reverse the protective effects of *B. infantis* (Fig. 3 D). This suggested that *B. infantis* inhibits GBS through upregulating PD-1.

## Discussion

Data obtained from clinical and animal model studies suggest that multi-lineage Th cells (Th1, Th2 and Th17) associated cytokine responses were elevated in the pathophysiology of GBS including IFN-γ, IL-4, IL-21 and IL-33 [25]. Regulatory T (Treg) cells are a special subpopulation of immunosuppressive T cells that are essential for sustaining immune homeostasis. Intravenous immunoglobulin (IVIg), the first line therapy in GBS, significantly promoted both the expansion of CD4^+^CD25^+^Foxp3^+^ regulatory T cells (Tregs) and secretion of IL-10 and TGF-β1 [26].

T lymphocytes are the main immune responsive cells. Its activation requires two signals. The first signal is the specific binding of T-cell surface receptors to the major histocompatibility complex of inflammatory cells. The second signal comes from costimulatory molecules, which can be divided into positive and negative costimulatory molecules according to different effects. Negative costimulatory molecules mainly prevent T cells from overstimulation, and PD-1/PD-L1 is a member of them, which acts as a deceleration and brake. After PD-1 is combined with PD-L1, PD-1 cytoplasmic immune receptor tyrosine phosphorylation leads to phosphorylation of multiple key molecular regions in the downstream TCR signaling pathway of T cell activation, and ultimately inhibits the proliferation and activity of T cells [5]. In addition, Ding et al. found that after PD-1/PD-L1 injected into EAN rats, the imbalance of Th1, Th2, Th17 and Treg cells in peripheral blood was significantly improved, which reduced the degree of autoimmune inflammation and sciatic nerve demyelination, reduced the course of EAN, and improved their clinical symptoms [17]. Therefore, the use of drugs to enhance PD-1/PD-L1 may provide a new idea for the clinical treatment of GBS.

PD-1 is a 288-amino acid type I transmembrane protein encoded by PDCD-1 gene and located on chromosome 2q37. It belongs to the CD28/cytotoxic T cell associated antigen 4(CTLA4) family. PD-L1 is a para-type transmembrane protein composed of 290 amino acids. It is encoded by CD274 gene on chromosome 9 [27]. PD-1 and its ligand, PD-L1, are expressed in activated T cells, B cells, natural killer cells, tumor-infiltrating lymphocytes and tumor cell surface to inhibit the immune function of T lymphocytes. The mechanism of autoimmune response is complex and involves multiple immune responses, among which PD-1/PD-L1 is one of the important mechanisms.

In the present study, in vitro and in vivo experiments were carried out to confirm that EAN animals exhibited obvious imbalance of Th1, Th2, Th17 and Treg cells. The protective role of *B.infantis* was related to PD-mediated immune regulation, thus inhibiting enlarged Th1/Th17 and promoting Treg. These results provide new ideas for early diagnosis and clinical treatment of GBS. The main limitations of this paper are a small number of patients. In addition, the number of animal models is small, and there are differences between animal models and actual pathology in human diseases.

## Conclusions

In the present study, in vitro and in vivo experiments were carried out to confirm the protective role of *B.infantis* was related to PD-mediated immune regulation, which provides new ideas for early diagnosis and clinical treatment of GBS.

## Supplementary Information

**Additional file 1.** PD-1 expression in vitro.

**Additional file 2.** GAPDH expression in vitro.

**Additional file 3.** PD-1 expression in vivo.

**Additional file 4.** GAPDH expression in vivo.

## Data Availability

The data are available from the corresponding author on reasonable request.
