# Supplementary figures and images for: B.infantis enhances immunotherapy for Guillain-Barre syndrome through regulating PD-1
Source: BMC Neurol. 2023 Jan 28;23:48. doi: 10.1186/s12883-022-03046-w (PMC9883859; doi:10.1186/s12883-022-03046-w)

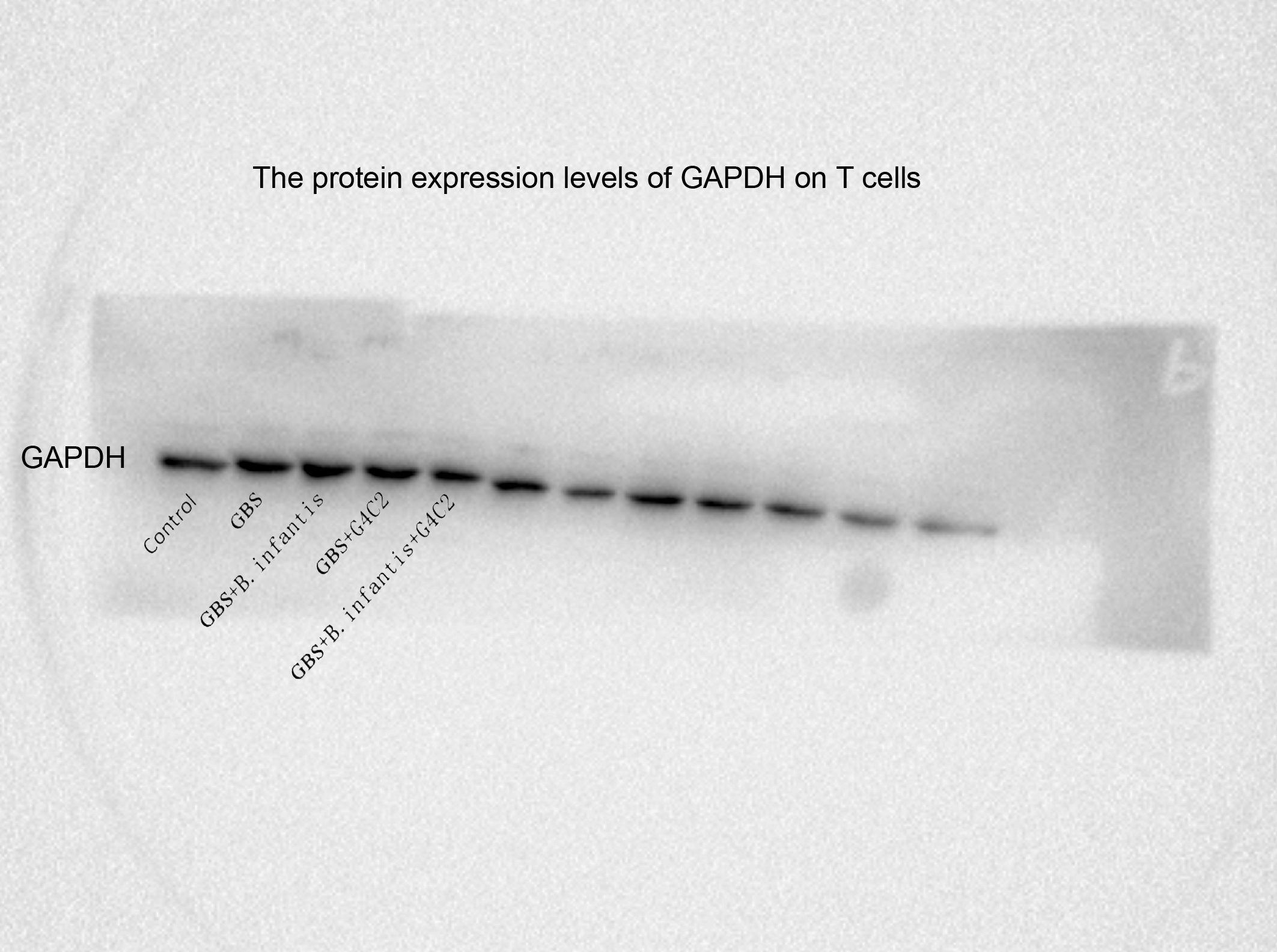

Supplement: Supplementary file 1 — Additional file 1. PD-1 expression in vitro. [file 12883_2022_3046_MOESM1_ESM.tif]

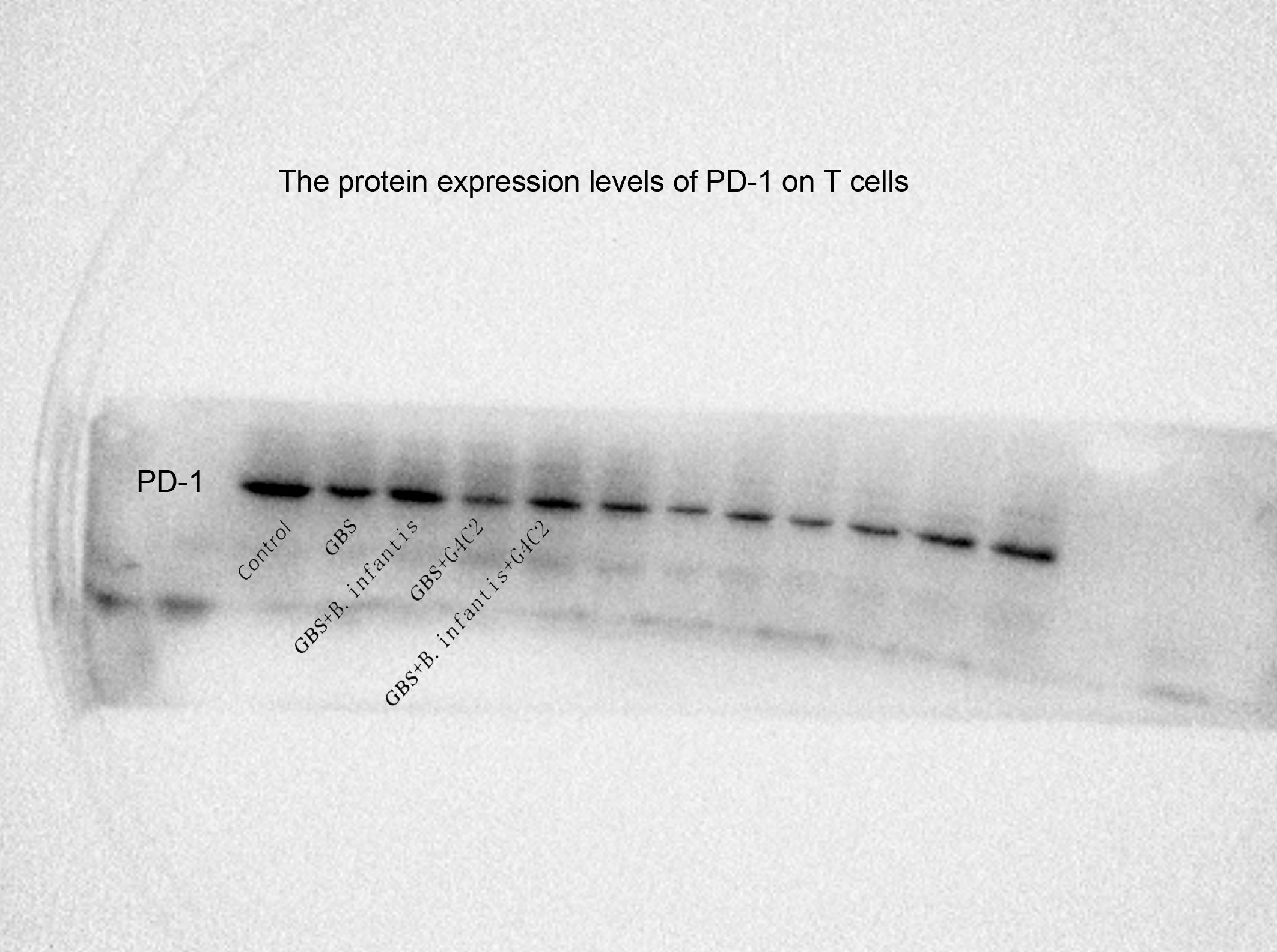

Supplement: Supplementary file 2 — Additional file 2. GAPDH expression in vitro. [file 12883_2022_3046_MOESM2_ESM.tif]

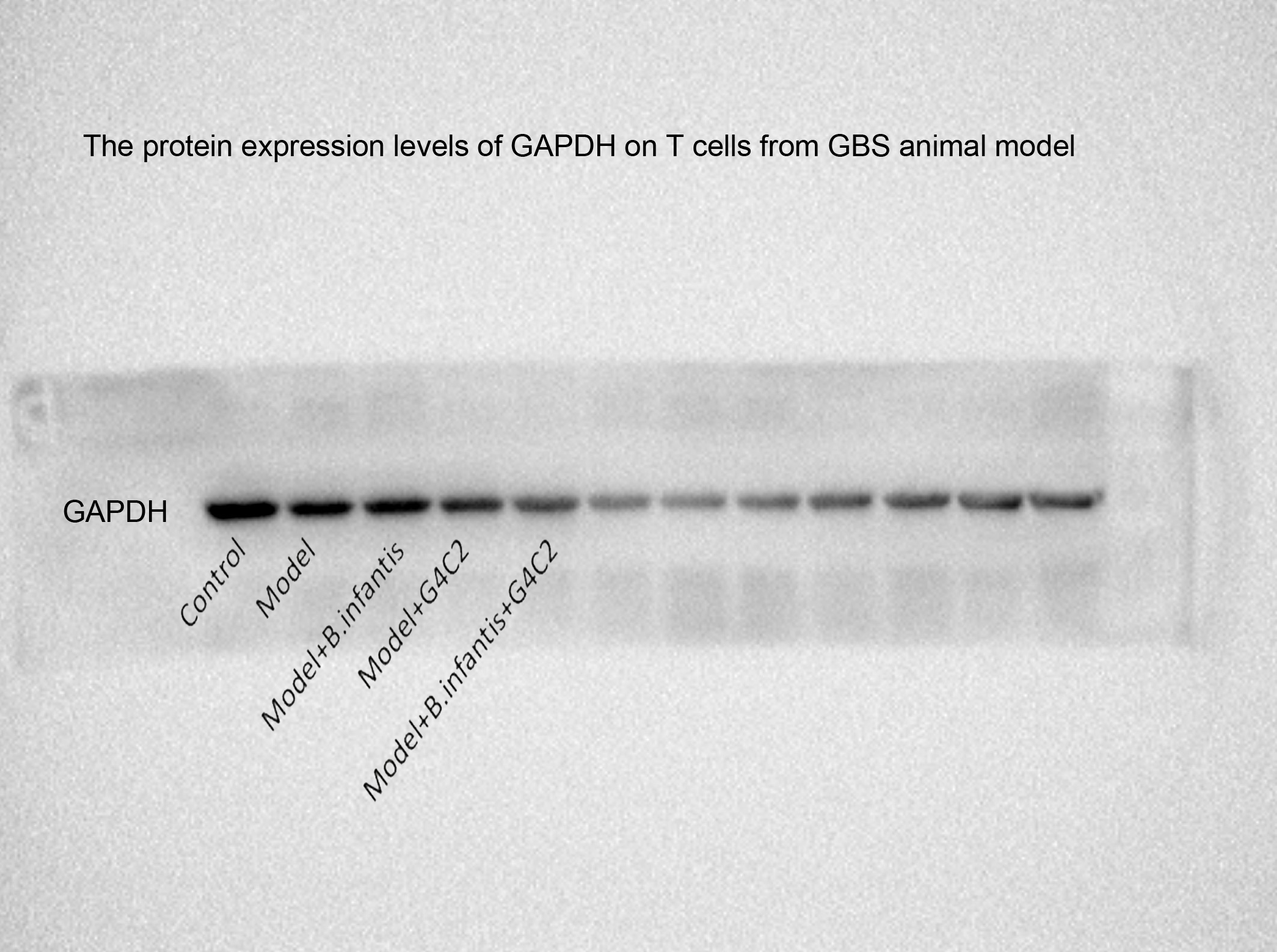

Supplement: Supplementary file 3 — Additional file 3. PD-1 expression in vivo. [file 12883_2022_3046_MOESM3_ESM.tif]

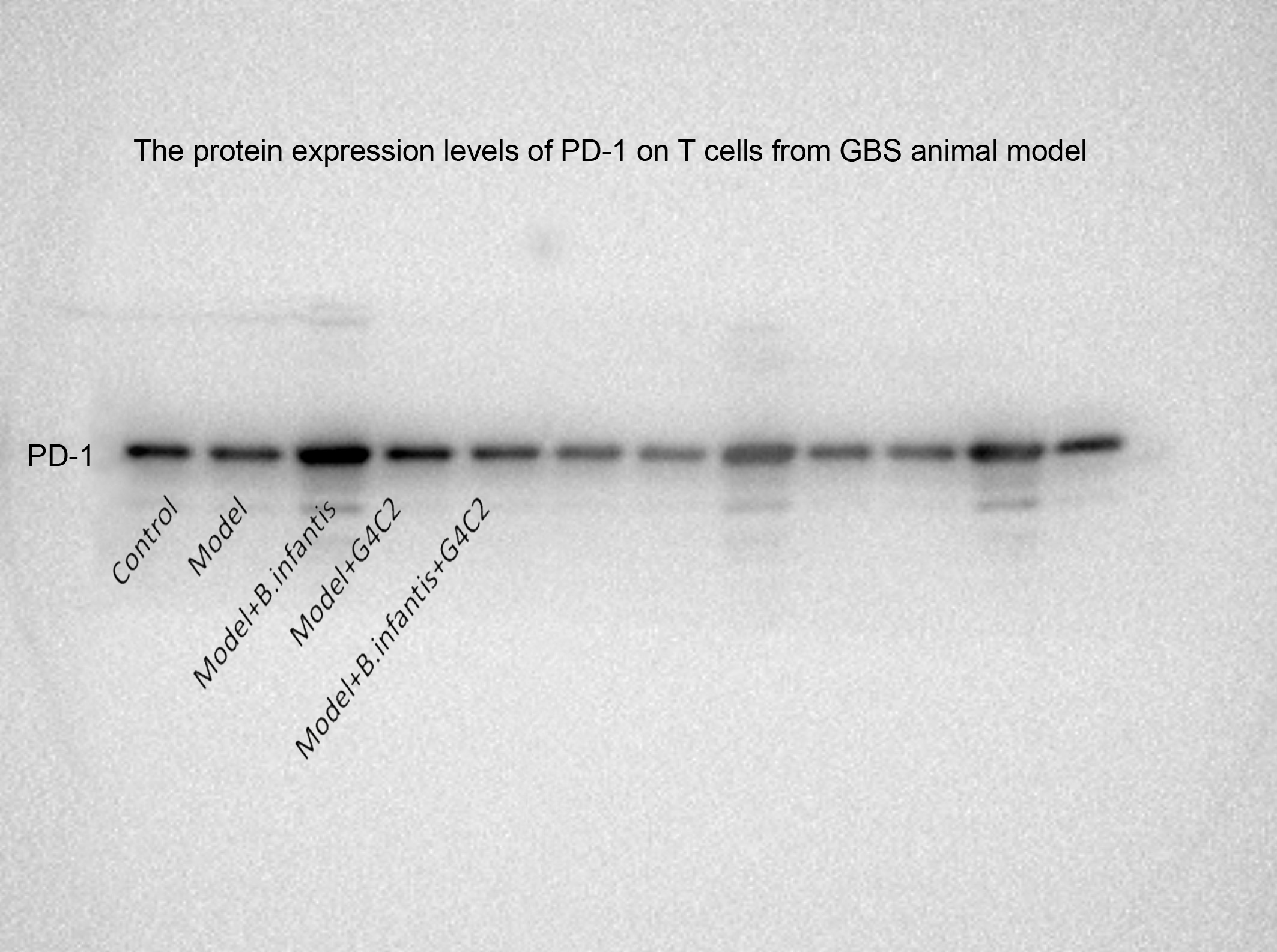

Supplement: Supplementary file 4 — Additional file 4. GAPDH expression in vivo. [file 12883_2022_3046_MOESM4_ESM.tif]
